# Validation of multiparametric MRI based prediction model in identification of pseudoprogression in glioblastomas

**DOI:** 10.1186/s12967-023-03941-x

**Published:** 2023-04-28

**Authors:** Laiz Laura de Godoy, Suyash Mohan, Sumei Wang, MacLean P. Nasrallah, Yu Sakai, Donald M. O’Rourke, Stephen Bagley, Arati Desai, Laurie A. Loevner, Harish Poptani, Sanjeev Chawla

**Affiliations:** 1 Radiology, Perelman School of Medicine at the University of Pennsylvania, Philadelphia, PA USA; 2 Clinical Pathology and Laboratory Medicine, Perelman School of Medicine at the University of Pennsylvania, Philadelphia, PA USA; 3 Neurosurgery, Perelman School of Medicine at the University of Pennsylvania, Philadelphia, PA USA; 4 Medicine, Perelman School of Medicine at the University of Pennsylvania, Philadelphia, PA USA; 5 Molecular and Clinical Cancer Medicine, University of Liverpool, Liverpool, UK

**Keywords:** Glioblastoma, Treatment response, Multiparametric MRI, Pseudoprogression, Diffusion MR imaging, Perfusion MR imaging

## Abstract

**Background:**

Accurate differentiation of pseudoprogression (PsP) from tumor progression (TP) in glioblastomas (GBMs) is essential for appropriate clinical management and prognostication of these patients. In the present study, we sought to validate the findings of our previously developed multiparametric MRI model in a new cohort of GBM patients treated with standard therapy in identifying PsP cases.

**Methods:**

Fifty-six GBM patients demonstrating enhancing lesions within 6 months after completion of concurrent chemo-radiotherapy (CCRT) underwent anatomical imaging, diffusion and perfusion MRI on a 3 T magnet. Subsequently, patients were classified as TP + mixed tumor (n = 37) and PsP (n = 19). When tumor specimens were available from repeat surgery, histopathologic findings were used to identify TP + mixed tumor (> 25% malignant features; n = 34) or PsP (< 25% malignant features; n = 16). In case of non-availability of tumor specimens, ≥ 2 consecutive conventional MRIs using mRANO criteria were used to determine TP + mixed tumor (n = 3) or PsP (n = 3). The multiparametric MRI-based prediction model consisted of predictive probabilities (PP) of tumor progression computed from diffusion and perfusion MRI derived parameters from contrast enhancing regions. In the next step, PP values were used to characterize each lesion as PsP or TP+ mixed tumor. The lesions were considered as PsP if the PP value was < 50% and TP+ mixed tumor if the PP value was ≥ 50%. Pearson test was used to determine the concordance correlation coefficient between PP values and histopathology/mRANO criteria. The area under ROC curve (AUC) was used as a quantitative measure for assessing the discriminatory accuracy of the prediction model in identifying PsP and TP+ mixed tumor.

**Results:**

Multiparametric MRI model correctly predicted PsP in 95% (18/19) and TP+ mixed tumor in 57% of cases (21/37) with an overall concordance rate of 70% (39/56) with final diagnosis as determined by histopathology/mRANO criteria. There was a significant concordant correlation coefficient between PP values and histopathology/mRANO criteria (r = 0.56; p < 0.001). The ROC analyses revealed an accuracy of 75.7% in distinguishing PsP from TP+ mixed tumor. Leave-one-out cross-validation test revealed that 73.2% of cases were correctly classified as PsP and TP + mixed tumor.

**Conclusions:**

Our multiparametric MRI based prediction model may be helpful in identifying PsP in GBM patients.

## Background

Glioblastoma (GBM) is a treatment-resistant and highly infiltrative primary brain neoplasm holding a devastating prognosis [1, 2]. Despite being treated with multimodal first-line treatment comprising of maximal safe surgical resection followed by concurrent chemo-radiotherapy (CCRT) along with adjuvant temozolomide (TMZ) [3, 4], the vast majority of patients (~ 80%) present a new contrasting-enhancing lesion in the radiation field within 6 months after the completion of CCRT [5–7]. This new lesion could be either true tumor growth, also known as tumor progression (TP), or predominant treatment effect, also known as pseudoprogression (PsP), which is mediated by TMZ induced increased vascular leakiness and intense immune response. The incidence of PsP ranges from 28 to 66% in GBM patients undergoing CCRT [8, 9].

The PsP lesions spontaneously subside or stabilize without a change in therapy, thus reflecting an effective outcome of CCRT [10, 11]. These PsP patients are usually surveilled with short-interval follow‐up magnetic resonance imaging (MRI) scans every 4–6 weeks and are treated with adjuvant TMZ. On the other hand, TP patients receive a second-line treatment, including repeat surgery and/or alternative therapies such as electric field therapy, immunotherapy, or anti-vascular therapy for potential benefits [12–14]. Thus, identifying patients with PsP is essential to avoid unwarranted repeat surgery, financial burden, and risky therapies. Confirming PsP is also valuable in preventing patients from being excluded from potentially effective experimental trials.

Conventional neuroimaging findings are often ambiguous in identifying PsP and present a significant diagnostic challenge [15, 16]. Therefore, there is a pressing need for the development of reliable, objective, and quantitative biomarkers for assessing treatment response in GBM patients. Using diffusion tensor imaging (DTI) and dynamic susceptibility contrast (DSC)-perfusion weighted imaging (PWI), some studies have reported sensitivities and specificities in the range of 62 -91% in distinguishing PsP from TP [17–22]. The variable degrees of success in these reports may be attributed to the fact that DTI and DSC-PWI were used independently in several of those studies, and imaging parameters were not integrated together to obtain a reliable discriminatory accuracy. On the other hand, multivariate regression analysis based prediction models are powerful tools that are frequently used in clinical practice to predict clinical outcomes [23]. Using a multiparametric analytical approach, we have previously developed a prediction model by combining the unique strengths of DTI and DSC-PWI derived parameters in differentiating PsP and TP with an accuracy of 91% in GBM patients who received surgery and CCRT [22].

To determine predictive power and prove its generalizability for broader applications in new populations of GBM patients across different clinical sites, it is essential to test the robustness of multiparametric based prediction model in evaluating treatment response. With this objective in mind, we sought to validate the findings of our previously established model [22] in a new, independent cohort of GBM patients treated with standard of care in identifying PsP in the present study.

## Materials and methods

### Patient population

This study was approved by the institutional review board and was compliant with the Health Insurance Portability and Accountability Act. The inclusion criteria for enrollment in the present study were that all patients had: (i) histologically confirmed diagnosis of GBM; (ii) treated with standard of care, i.e. surgery and CCRT, (iii) exhibited new enhancing lesion in the radiation field on follow-up MRI within 6 months after completion of CCRT, (iv) had the availability of anatomical and physiological neuroimaging data (DTI and DSC-PWI). Based upon the inclusion criteria, a cohort of 56 patients (25 females/31 males; mean age: 61.2 ± 9.4 years) was recruited in the present study.

These patients were grouped into two categories: PsP (n = 19) and TP + mixed tumor (n = 37). Patients in whom tumor specimen was available from repeat surgery/biopsy, malignant features on histopathology were used to identify PsP (< 25% malignant features; n = 16) and TP + mixed tumor (> 25% malignant features; n = 34) [24, 25]. In the case of non-availability of tissue specimens,  ≥2 consecutive follow-up standard-of-care MRI scans using mRANO criteria [26] were used to determine the status of PsP (n = 3) or TP + mixed tumor (n = 3). The final diagnosis for each patient as PsP or TP + mixed tumor was established by a consensus opinion at a weekly multidisciplinary neuro‐oncology conference.

### Data acquisition

All Patients underwent MRI on a 3 T Tim Trio whole body MR scanner (Siemens, Erlangen, Germany) equipped with a 12-channel phased array head coil. The anatomical imaging protocol included axial 3D-T1-weighted magnetization-prepared rapid acquisition of gradient echo (MPRAGE) imaging and an axial T2-FLAIR imaging using standard parameters. The postcontrast T1-weighted images were acquired with the same parameters as the precontrast acquisition after administration of standard dose (0.14 mmol/Kg) of gadobenate dimeglumine (MultiHance, Bracco Imaging, Milano, Italy) intravenous contrast agent using a power injector (Medrad, Idianola, PA).

### Diffusion tensor imaging

Axial DTI data were acquired using 30 noncollinear/noncoplanar directions with a single-shot spin-echo, echo-planar read-out sequence with parallel imaging by using generalized autocalibrating partially parallel acquisition (GRAPPA) and acceleration factor of 2. The sequence parameters were as follows: repetition time (TR) / echo time (TE) = 5000/86 ms, number of excitations (NEX) = 3, field of view (FOV) = 22 × 22cm^2^, matrix size = 128 × 128, in-plane resolution = 1.72 × 1.72 mm^2^; slice thickness = 3 mm; b = 0, 1000 s/mm^2^; number of slices = 40; acquisition time 8 min.

### Dynamic susceptibility contrast-perfusion weighted imaging

For axial DSC-PWI, a bolus of gadobenate dimeglumine (Multi-Hance; Bracco Diagnostics, Princeton, New Jersey) was injected with a preloading dose of 0.07 mmol/kg, to reduce the effect of contrast agent leakage on CBV measurements. A T2*-weighted gradient-echo EPI was used during the second 0.07 mmol /kg bolus of contrast agent for the DSC-PWI. The injection rate was 5 ml/s for all patients and was immediately followed by a flush of saline (total of 20 ml at the same rate). The sequence parameters were as follows: TR/TE = 2000/45 ms; FOV = 22 × 22 cm^2^; matrix size = 128 × 128; in-plane resolution = 1.72 × 1.72 mm^2^; slice thickness = 3 mm; bandwidth = 1346 Hz/pixel; flip angle = 90°; EPI factor = 128; echo spacing = 0.83; acquisition time 3 min and 10 s. Forty-five sequential measurements were acquired for each section.

### Image processing and data analysis

The motion and eddy current correction algorithms were applied to raw DTI data using in-house developed algorithm (IDL; ITT Visual Information Solutions, Boulder, Colorado). Pixel-wise mean diffusivity (MD), fractional anisotropy (FA), coefficient of linear anisotropy (CL), planar anisotropy (CP), and spherical anisotropy (CS) maps were computed by using the methods reported previously [27, 28]. Leakage-corrected cerebral blood volume (CBV) maps were generated by performing gamma-variate curve fitting from DSC-PWI data using NordicICE software (NordicNeuroLab, Bergen, Norway).

The DTI derived maps, CBV maps, and T2-FLAIR images were resliced and co-registered to post-contrast T1-weighted images. A semiautomatic approach was used to segment the contrast-enhancing regions of each lesion by using a signal intensity-based thresholding method [27, 28]. The median values of DTI metrics (MD, FA, CL, CP, and CS) from the enhancing regions were computed. The CBV values from the enhancing regions were normalized by corresponding values from contralateral normal white matter regions to obtain relative CBV (rCBV). The top 90th percentile rCBV values were also measured from the enhancing regions and were reported as rCBV_max_.

### Radiographic response assessment using mRANO criteria

Those patients in whom repeat surgery or biopsy was not possible, a well-established mRANO criteria [26] were used to determine the final diagnosis of PsP and TP + mixed response by a board-certified neuroradiologist (SM). The tumor size was determined as the sum of the products of diameters (SPD) on the post-contrast T1 images. As the mRANO working group has suggested that radiological response at the initial presentation should persist for at least 4 weeks on follow-up imaging before it can be considered as PsP or TP, tumor size was measured again at the follow-up scan [26].

### Response assessment using histological/immunohistochemical analysis

Tumor specimens were originally cut, mounted, and stained with hematoxylin-eosin (H & E). Immunohistochemistry for Ki-67 and p53 was performed by using a Bond III automated system (Leica Biosystems, Buffalo Grove, Illinois). The entire submitted material for each case was examined by a board-certified neuropathologist (MPN), who was blinded to the results of the MR imaging studies. The slides were examined to determine the relative degree of recurrent glioma and treatment-related changes by standard methods. The proliferative index of Ki-67 for each case was calculated as a percentage of positive tumor cells, avoiding areas of inflammatory infiltrates [29].

### Validation of multiparametric MRI based prediction model

In our previous study [22], GBM patients presenting with new enhancing lesions within 6 months of completing CCRT were classified into two groups, TP group (malignant features > 75%), and PsP + mixed tumor [PsP (malignant features < 25%) combined with mixed tumors (malignant features = 25–75%)], based on histopathological findings from tumor specimens obtained from repeat surgery. Significantly elevated FA, CP, CL, and rCBV_max_ values were observed in TP compared to those with PsP + mixed tumor from contrast-enhancing regions of neoplasms with variable sensitivities (62–71%) and specificities (75–90%) of individual parameters. However, the best prediction model in differentiating TP from PsP + mixed response was obtained when FA, CL, and rCBV_max_ were incorporated into the multivariate logistic regression analyses. The receiver operative characteristic (ROC) curve revealed an accuracy of 91% in distinguishing TP from PsP + mixed tumor. In the present study, a combination of these three parameters (FA, CL, and rCBV_max_) was used to compute the predictive probabilities (PP) of TP for each lesion using the following regression equation:

$${f\left( {FA,CL, rCBVmax } \right) = 1 \div 1 + \exp \left( { - \left( {\beta_0 + \beta_1FA + \beta_2CL + \beta_3rCBVmax } \right)} \right),}$$

where *β0* = − 16.17, *β1* = 194.01, *β2* = − 285.65, and *β3* = 1.21.

**Fig. 1:**
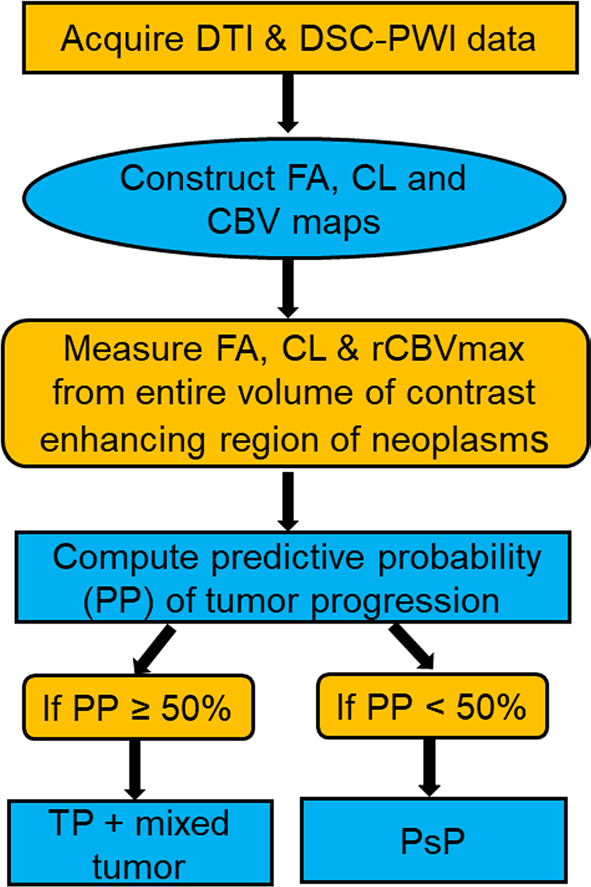
The step-wise process of utilizing multiparametric MRI based prediction model in determining PsP and TP+mixed tumors

As the recent evidence [24, 25] has suggested that the presence of malignant features of less than 25% within the tumor specimens from repeat surgery after the completion of CCRT in GBMs does not usually alter the clinical management of these patients, and these patients are continued on adjuvant TMZ treatment, patients were classified into two modified groups. PsP group (malignant features < 25%) and TP + mixed tumor (malignant features > 25%). In the next step, the PP values were used to characterize each enhancing lesion as PsP or TP + mixed tumor. The lesions were considered as PsP if the PP was < 50% and TP + mixed tumor if the PP was ≥ 50%. The step-wise process of utilizing multiparametric MRI based prediction model in determining PsP and TP + mixed tumors is presented as a flow chart in Fig. 1.

### Statistical analysis

Kolmogorov-Smirnov tests were used to determine the nature of data distribution. As the data showed a departure from Gaussian distribution, non-parametric Mann-Whitney U tests were performed to assess differences in the median values of FA, CL, and rCBV_max_ between TP + mixed response and PsP groups as determined by histopathology/mRANO criteria. A probability (p) value of less than 0.05 was considered significant.

We sought to ascertain the number of cases correctly classified as PsP or TP + mixed tumor using multiparametric MRI-based prediction model using histopathology/mRANO criteria as ground truth in the final diagnosis of PsP and TP + mixed tumor. Pearson test was used to determine the concordance correlation coefficient between PP values and histopathology/mRANO criteria to ascertain the robustness of our prediction model. The area under the ROC curve (AUC) was used as a quantitative measure for assessing the discriminatory accuracy of prediction model in identifying PsP and TP + mixed response. Additionally, a leave-one-out cross-validation analysis was applied to estimate model’s potential to predict outcomes in a new independent data set. All statistical analyses were performed using a statistical package, SPSS for Windows (v. 18.0; Chicago, IL).

## Results

**Fig. 2:**
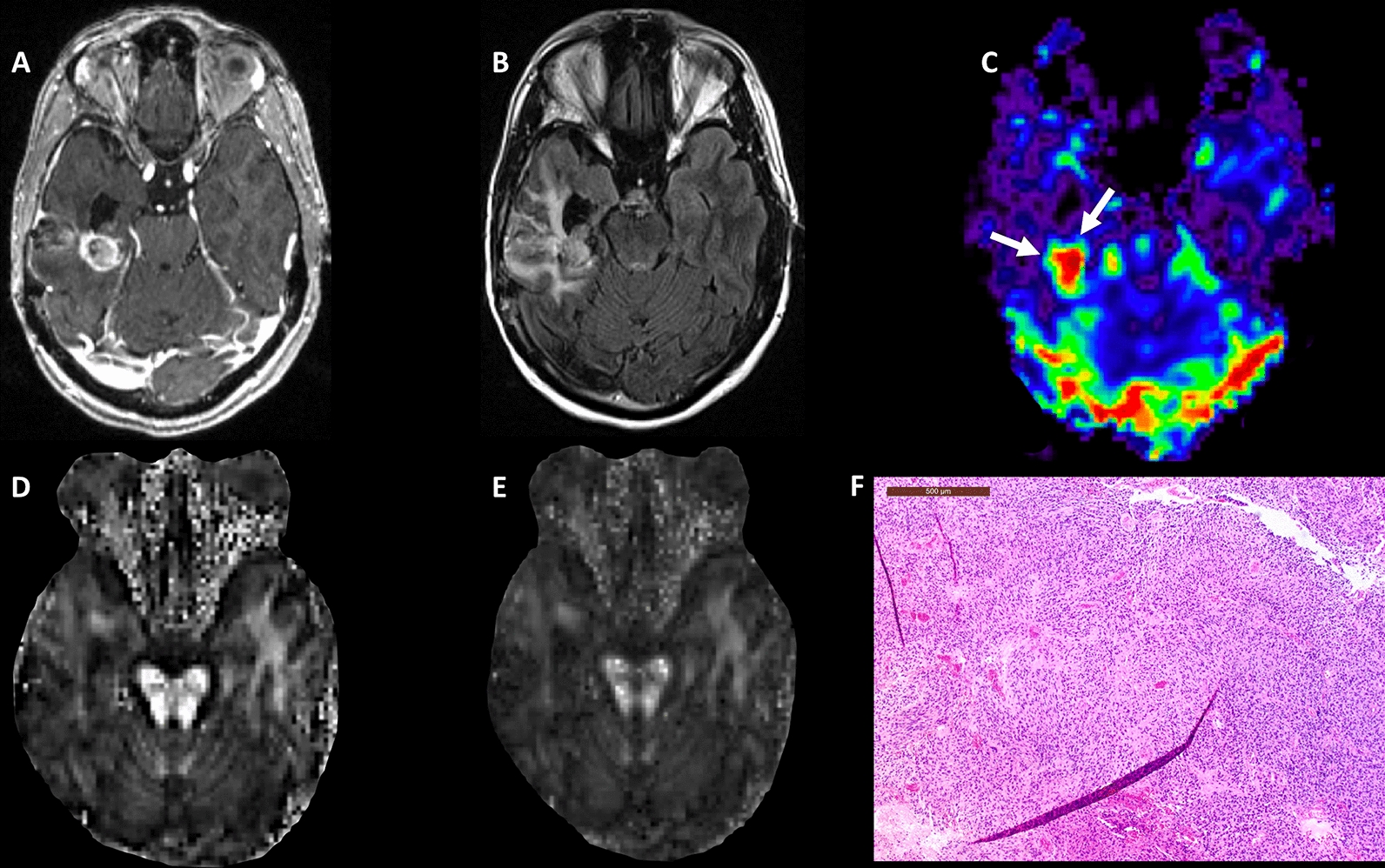
A 61-year-old male patient with glioblastoma, status post gross total resection and chemoradiation. **A** Post-contrast T1-weighted image shows a heterogeneously enhancing lesion at the site of previously resected glioblastoma, which had increased from prior scans. **B** T2-FLAIR image demonstrates hyperintense signal abnormality surrounding the lesion. **C** DSC-PWI shows elevated rCBV from the posterior enhancing region of the tumor (white arrows). Overall the constellation of these conventional and advanced imaging findings was concerning for true progression. Multiparametric MRI based prediction model comprising rCBV_max_, FA **D**, and CL **E** revealed a diagnosis of TP+mixed tumor (rCBV_max_ = 7.93, FA = 0.15, CL = 0.05), suggesting a significant component of recurrent tumor (PP = 84%). **F** The hematoxylin and eosin stained sections demonstrate hypercellular tissue representative of tumor with hyperchromatic irregular nuclei, and minimal treatment-related changes

**Fig. 3:**
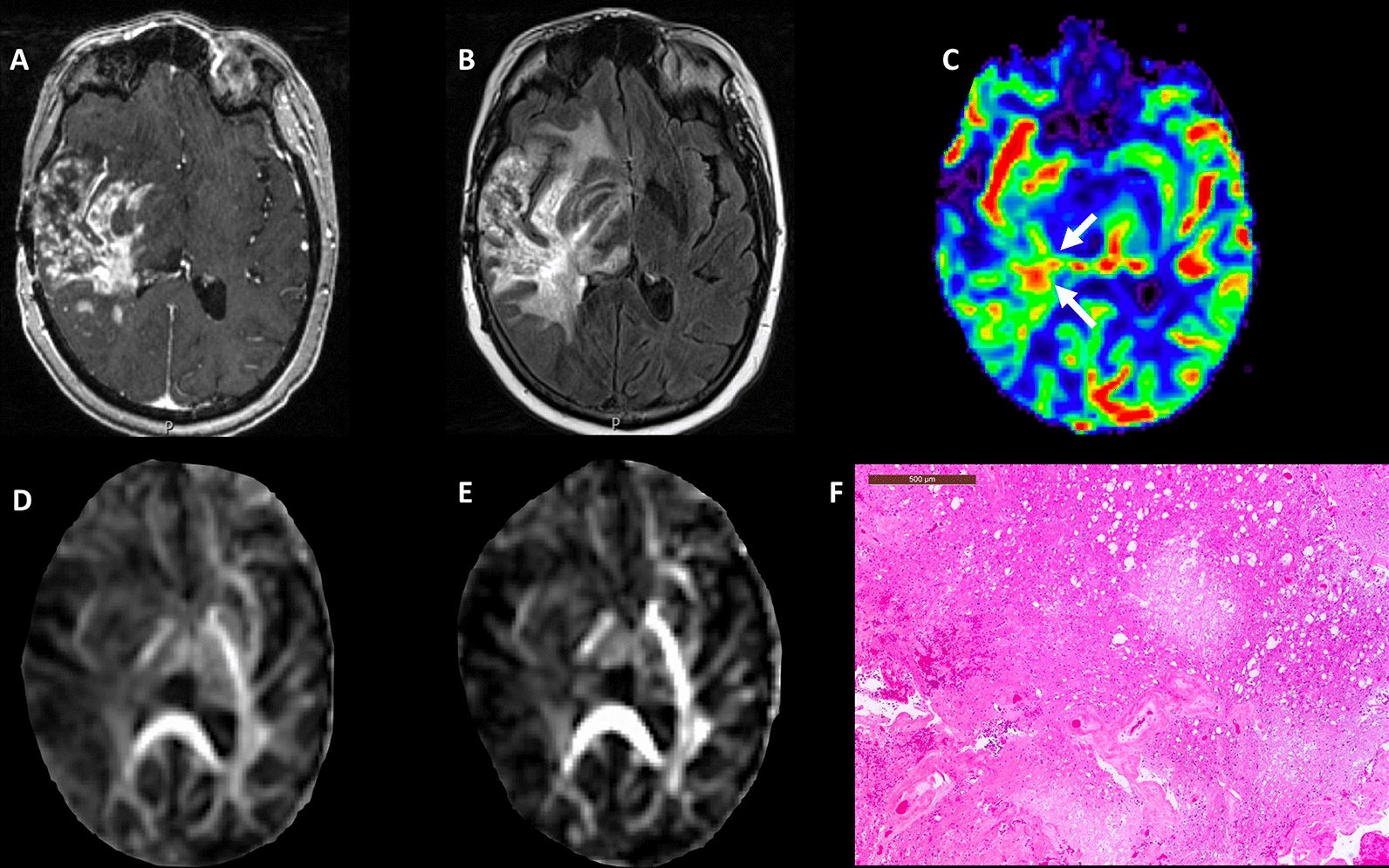
A 63-year-old male patient with glioblastoma, status post gross total resection and chemoradiation. **A** Post-contrast T1-weighted image shows a heterogeneously enhancing lesion at site of previously resected glioblastoma which had increased from prior scans. **B** T2-FLAIR images demonstrate associated hyperintense signal abnormality surrounding the lesion. **C** DSC-PWI shows mildly elevated rCBV from the enhancing region of the tumor. Overall constellation of these conventional and advanced imaging findings was concerning for true progression. Multiparametric MRI based prediction model comprising rCBV_max_ with FA **D** and CL **E** revealed a diagnosis of PsP (rCBV_max_ = 2.61, FA = 0.13, CL = 0.05), suggesting a significant component of treatment-related changes (PP = 19%). **F** The hematoxylin and eosin stained sections show infarct, abundant macrophages, and hyalinized vessels, indicative of treatment-related changes

**Fig. 4:**
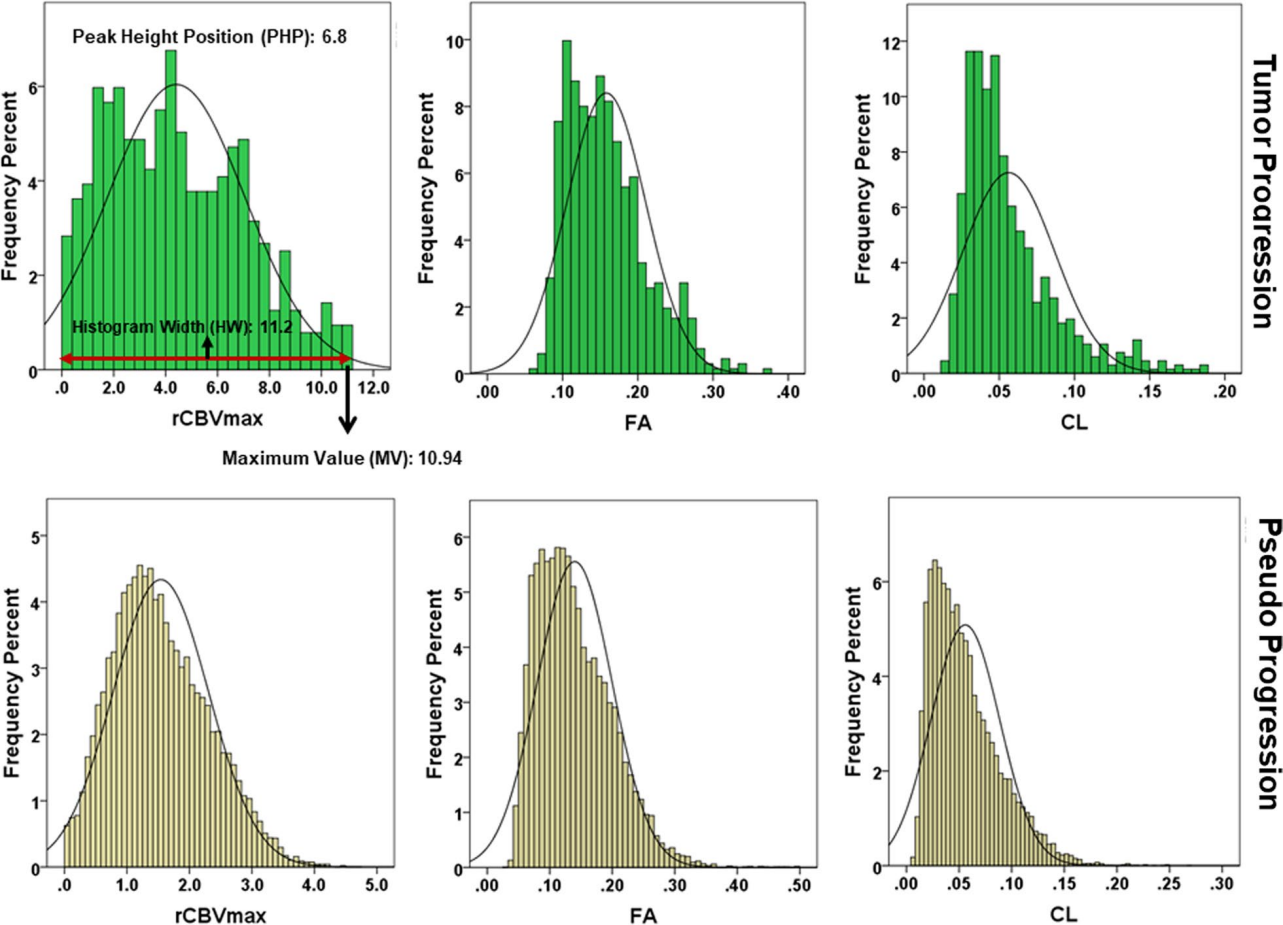
Histograms showing the frequency distributions of parameters (FA, CL and rCBV) from entire volume of contrast-enhancing regions of neoplasms from two patients shown in Figs. 2 and 3. Please note the presence of greater peak height positions (PHP), histogram widths (HW) and maximum pixel values (MV) of these parameters in TP than in PsP

**Fig. 5:**
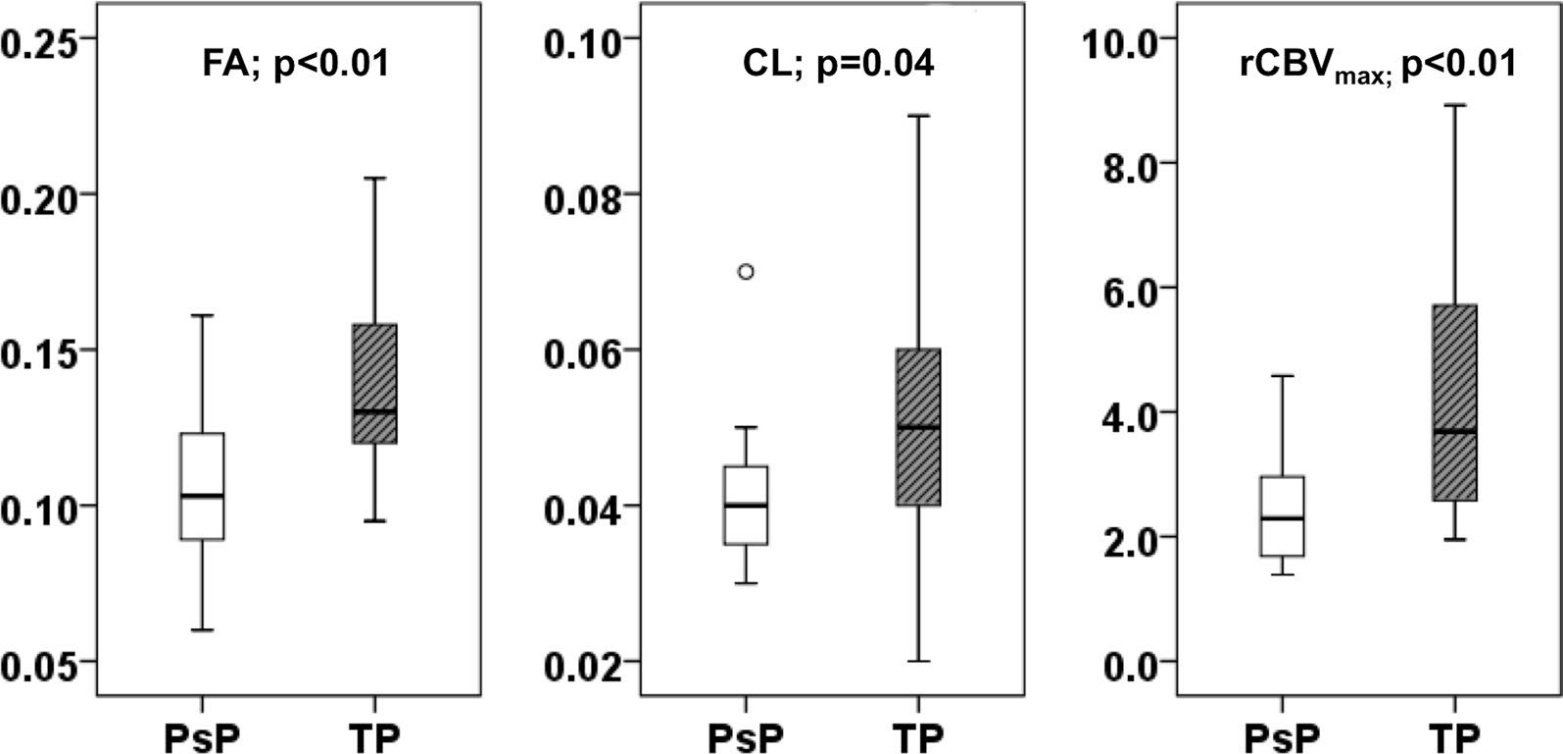
Box‐and‐whisker plots demonstrating the distribution of diffusion (FA and CL) and perfusion (rCBV_max_) parameters from contrast enhancing regions of neoplasms in TP+mixed tumor (gray), and PsP (white) patients. The *solid line* inside each box represents the median value, while the edges represent the 25th and 75th percentiles. The straight line (bars) on each box indicates the range of data distribution. *Circles* represent outliers (values 1.5 box length from the 75th/25th percentiles)

Representative anatomical images, DTI derived parametric maps (FA, CL), and CBV maps each from a patient with TP + mixed tumor and PsP are shown in Figs. 2 and 3, respectively. Figure 4 demonstrates the histogram distributions of parameters (FA, CL and rCBV) encompassing the entire volume of contrast-enhancing lesions from two patients shown in Figs. 2 and 3. The distributions of FA, CL, and rCBV_max_ values from contrast-enhancing lesions of all patients are shown as box-and-whisker plots (Fig. 5). Significantly higher FA (mean ± standard deviation = 0.14 ± 0.03 vs. 0.11 ± 0.02, p < 0.01); CL (0.05 ± 0.03 vs. 0.04 ± 0.01, p = 0.04) and rCBV_max_ (4.09 ± 1.85 vs. 2.49 ± 0.97, p < 0.01) values were observed in TP + mixed tumors than in PsP patients.

**Fig. 6:**
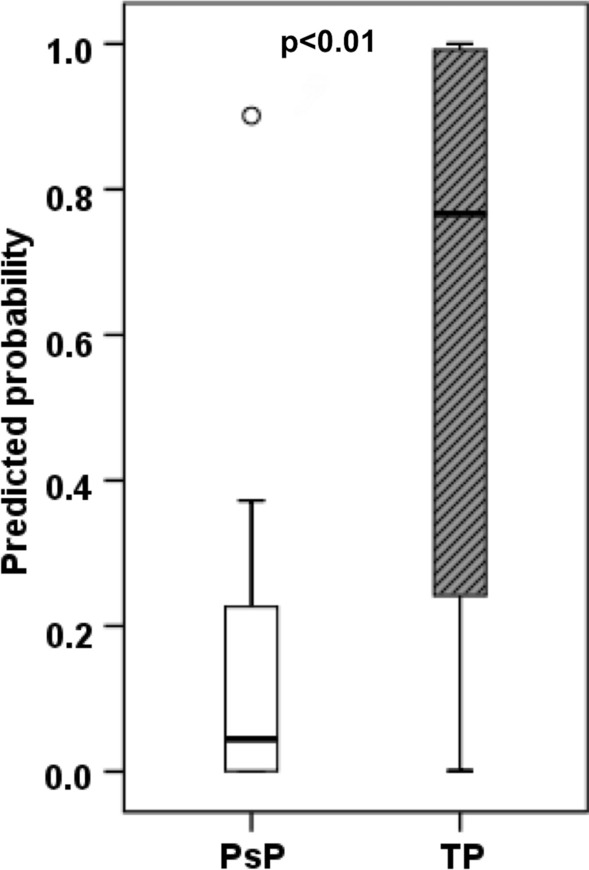
Box‐and‐whisker plots demonstrating the distribution of multiparametric MRI derived predicted probabilities (PP) of tumor progression between PsP and TP+mixed tumor patients. The bottom and top edges of boxes represent the 25th percentile and the 75th percentile values. The bands within the boxes represent 50th percentile (median) values. Whiskers display the range of data distribution. Circles represent outliers (values 1.5 box length from the 75th/25th percentiles)

While characterizing each lesion as PsP or TP + mixed tumors, our prediction model correctly predicted PsP in 95% (18/19) and TP + mixed tumor in 57% of cases (21/37) with an overall concordance rate of 70% (39/56) with the final diagnosis as determined by histopathology/mRANO criteria. Additionally, a significant concordant correlation coefficient between PP values and histopathology/mRANO criteria (r = 0.56; p < 0.001) was observed. As shown in Fig. 6, PP values were significantly higher in TP + mixed tumors (mean = 61.3 ± 39.1%) than in PsP cases (mean = 15.1 ± 22.2%) with a p-value of  < 0.01.

**Fig. 7:**
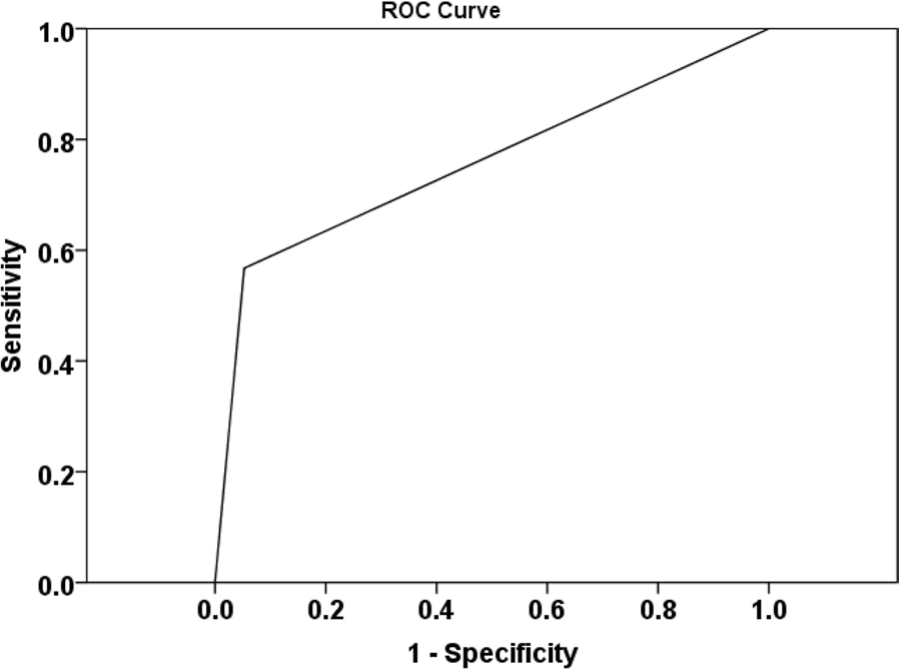
ROC curve from a combination of imaging parameters (FA, CL, and rCBV_max_) provided an AUC of 0.757 in identifying PsP and TP+mixed tumors. Leave-one-out cross-validation test revealed that 73.2% of cases were correctly classified as PsP and TP+mixed tumors

The ROC analyses revealed an accuracy of 75.7% in distinguishing PsP from TP + mixed tumors (Fig. 7). Leave-one-out cross-validation test revealed that 73.2% of cases were correctly classified as PsP and TP + mixed tumors.

## Discussion

Conducting a validation study is essential for providing a realistic estimate of predictive power of a previously developed model derived from one data set that would occur when applied to a new sample [30]. In the present study, we validated our previously developed multiparametric MRI-based prediction model [22] in identifying PsP in a new, independent cohort of GBM patients treated with CCRT and presenting with a contrast enhancing lesion within 6 months following treatment. A significant concordant correlation coefficient was observed between multiparametric MRI derived PP values and histopathology/mRANO criteria for determining the final diagnosis of PsP and TP + mixed tumor. Our multiparametric MRI-based prediction model correctly classified enhancing lesions as PsP or TP + mixed tumor in 70% of the new cases. We believe that this is a very promising finding given the fact that GBMs are extremely heterogeneous neoplasms in nature, both phenotypically and genotypically, and this intratumoral heterogeneity increases even further in the posttreatment settings [31, 32].

An important finding was that 95% of PsP cases were correctly classified by our prediction model. The incidence of PsP ranges from 28 to 66% in GBM patients undergoing CCRT [33]. Accurate stratification of PsP cases may be helpful in preventing these patients from getting unnecessary aggressive neuro-interventional procedures such as repeat surgery or biopsy. Moreover, PsP patients are more responsive to TMZ treatment and tend to have better clinical outcomes than TP patients. Therefore, PsP patients are clinically managed with continuation of adjuvant TMZ and closely monitored with short interval follow-up MRI scans [5].

The combination of spatial and temporal tumor heterogeneities present within GBMs can cause local variations in the physiological features such as cellularity, vascularity, and metabolic activities, which are reflected by mismatch in the findings from diverse neuroimaging parameters. Therefore, the usage of a single imaging modality or parameter may not always be appropriate in characterizing GBMs with high accuracy [11]. In such situations, integrated analytical approach of combining of DTI and DSC-PWI sensitive parameters may allow more reliable assessment of tumor biology and microenvironment. Indeed, the multiparametric MRI approach has shown great potential in differentiating necrotic GBMs from brain abscesses [28], identification of histologic grades of gliomas [34], differential diagnosis of brain neoplasms [35, 36], discrimination of recurrent tumors from radiation necrosis [37], investigation of tumor invasion [38], prognostication [39], and evaluation of treatment response to immunotherapy [40] and targeted therapy [41] in GBM patients.

Using multiparametric data analytical method, we used a combination of three parameters (FA, CL, and rCBV_max_) in developing a classification model for distinguishing PsP + mixed tumor from TP in GBM patients in our previous study [22]. The ROC analysis revealed a high discriminatory accuracy of 91%. When PP values derived from a combination of FA, CL, and rCBV_max_ were used in identifying PsP from a new cohort of GBM patients in the present study, a moderate discriminatory accuracy of 75.7% was observed using the ROC analysis. The possible explanation for this decrease in the validity of our prediction model in the new data set might be due to the use of different categorization of patients [PsP and TP + mixed tumor in the present study vs. TP and PsP + mixed tumor in our previous study [22]]. The rationale for regrouping the patients lies in the fact that patients showing mixed tumor (25–75% malignant features) are subjected to repeat surgery and/or to alternate therapies such as tumor treating fields or immunotherapy in a similar manner as those with TP (> 75% malignant features) in the usual clinical practice [24, 25]. Therefore, keeping best clinical practice in mind and for appropriate treatment stratification, clustering of patients showing mixed tumor to those with TP in the same group seems more appropriate.

Our findings should be treated with caution as our multiparametric MRI based prediction model achieved a moderate discriminatory accuracy (75.7%) in differentiating PsP from TP + mixed tumor. We believe that our prediction model can be further improved by combined analysis of DTI and DSC-PWI data both from contrast enhancing and peritumoral regions of neoplasms along with incorporation of molecular information such as MGMT promoter methylation and/or isocitrate dehydrogenase (*IDH*) mutational status into the multivariate regression analysis in the future studies.

In conclusion, our multiparametric MRI based prediction model allows quantitative, and objective evaluation of treatment response in GBM patients. Our results indicate that this prediction model may be helpful in identifying PsP cases. Recognizing patients with PsP is critical for prognostication and for guiding clinical decision making.

## Data Availability

The analyzed data are available from the corresponding author on reasonable request.

## Abbreviations

CCRT: Concurrent chemo-radiotherapy
CL: Coefficient of linear anisotropy
CP: Coefficient of planar anisotropy
CS: Coefficient of spherical anisotropy
DSC-PW1: Dynamic susceptibility contrast-perfusion weighted imaging
DTI: Diffusion tensor imaging
FA: Fractional anisotropy
GMB: Glioblastoma
MD: Mean diffusivity
MRI: Magnetic resonance imaging
PP: Predictive probability
PSP: Pseudoprogression
rCBV_max_: Maximum relative cerebral blood volume
TMZ: Temozolomide
TP: True progression
